# Evolution of guanylate binding protein genes shows a remarkable variability within bats (Chiroptera)

**DOI:** 10.3389/fimmu.2024.1329098

**Published:** 2024-01-31

**Authors:** Ana Pinheiro, J. Ricardo Borges, João Vasco Côrte-Real, Pedro J. Esteves

**Affiliations:** ^1^ CIBIO-UP, Centro de Investigação em Biodiversidade e Recursos Genéticos, Universidade do Porto, InBIO, Laboratório Associado, Vairão, Portugal; ^2^ BIOPOLIS Program in Genomics, Biodiversity and Land Planning, CIBIO, Vairão, Portugal; ^3^ Departamento de Biologia, Faculdade de Ciências, Universidade do Porto, Porto, Portugal; ^4^ Max von Pettenkofer Institute and Gene Center, Virology, National Reference Center for Retroviruses, Faculty of Medicine, Ludwig Maximilian University of Munich (LMU) München, Munich, Germany; ^5^ CITS - Centro de Investigação em Tecnologias de Saúde, CESPU, Gandra, Portugal

**Keywords:** GBP, innate immunity, evolution, bats, genome, birth and death model of evolution

## Abstract

**Background:**

*GBP*s (guanylate binding proteins), an evolutionary ancient protein family, play a key role in the host’s innate immune response against bacterial, parasitic and viral infections. In Humans, seven *GBP* genes have been described (*GBP1-7*). Despite the interest these proteins have received over the last years, evolutionary studies have only been performed in primates, *Tupaia* and rodents. These have shown a pattern of gene gain and loss in each family, indicative of the birth-and-death evolution process.

**Results:**

In this study, we analysed the evolution of this gene cluster in several bat species, belonging to the Yangochiroptera and Yinpterochiroptera sub-orders. Detailed analysis shows a conserved synteny and a gene expansion and loss history. Phylogenetic analysis showed that bats have *GBP*s *1*,*2* and *4*-*6*. *GBP2* has been lost in several bat families, being present only in Hipposideidae and Pteropodidae. *GBP*s*1*, *4* and *5* are present mostly as single-copy genes in all families but have suffered duplication events, particularly in *Myotis myotis* and *Eptesicus fuscus.* Most interestingly, we demonstrate that *GBP6* duplicated in a Chiroptera ancestor species originating two genes, which we named *GBP6a* and *GBP6b*, with different subsequent evolutionary histories. *GBP*6a underwent several duplication events in all families while *GBP*6b is present as a single copy gene and has been lost in Pteropodidae, Miniopteridae and *Desmodus rotundus*, a Phyllostomidae. With 14 and 15 *GBP* genes, *Myotis myotis* and *Eptesicus fuscus* stand out as having far more copies than all other studied bat species. Antagonistically, Pteropodidae have the lowest number of *GBP* genes in bats.

**Conclusion:**

Bats are important reservoirs of viruses, many of which have become zoonotic diseases in the last decades. Further functional studies on bats *GBP*s will help elucidate their function, evolutionary history, and the role of bats as virus reservoirs.

## 1 Introduction

Guanylate-binding proteins (*GBP*s) are a family of evolutionary ancient, conserved proteins that have vital roles in host defence against intracellular pathogens, ranging from viruses to bacteria (1, 2). These proteins belong to the large dynamin guanosine triphosphatases (GTPases) superfamily and the IFN-inducible guanosine triphosphatases (3) and share structural and biochemical similarities (3–5). The structure of these proteins comprises a globular N-terminal large GTPase (LG) domain connected by a hinge region to the middle domain (MD) and the GTPase effector domain (GED) at the C-terminus. The LG domain is involved in GTPase and GDPase activity and Mg2+ cofactor finding [reviewed in (3, 6)]. *GBP* expression is triggered by inflammatory signals, the most potent of which are interferons (IFN), but also interleukins (IL) and tumour necrosis factor (TNF) [reviewed in (3)]. As such, they are part of the cell-autonomous innate immune response and have been considered major players in the host’s innate immunity.

In mammals, *GBP*s are usually organized in tandem in one chromosome (5, 7) but in some rodents, like *Mus musculus* and *Rattus norvegicus*, *GBP*s are organized in two different gene clusters (8). Surprisingly, this gene family was only studied in primates, *Tupaia* and rodents (8–10). The evolutionary history of *GBP*s is complex, with duplications, deletions and neofunctionalization of genes as expected in gene families following the birth-and-death model of evolution (11). The number of *GBP* genes varies among species; not all *GBP* orthologs are present in every species and some are limited to a specific mammalian group. Seven *GBP*s have been described in humans, *GBP*s1-7 (1, 5). Of these, *GBP*3 seems to have emerged through a duplication of *GBP*1 in Simiiformes gaining a new function, regulation of caspase-4 activation (12). *GBP*7 most likely emerged from a duplication of *GBP*4 in primates being specific to this group (9). Muroids (Rodentia) share *GBP2*, *GBP5* and *GBP6* orthologs with primates and furthermore have four exclusive *GBPs*, *GBPa-d*. Each of the seven Muroid *GBP*s has its own pattern of duplications and deletions (8). In *Tupaia*, five *GBP*s have been described: *GBP1*, *GBP2*, *GBP4*, *GBP5* and *GBP7*, all seemingly orthologs to primates’ *GBP*s except for *GBP7* (10).

Bats belong to the order Chiroptera, the second largest mammalian order after Rodentia and have adapted to diverse ecological niches across the planet (13). The Chiroptera radiation occurred approximately 60 million years ago (mya) (14–16). Based on molecular genetics data, the order Chiroptera is subdivided into two suborders, Yangochiroptera (composed exclusively of microbat families) and Yinpterochiroptera (composed of five microbat families and all megabat families) (14). Bats are known reservoirs of many viruses in the animal kingdom, and have been assigned as the source of many human viral diseases in modern times, including SARS-CoV (China, 2002/2003) (17), Marburg virus (Africa, 2005) (18), MERS-CoV (Middle East, 2012) (19, 20), Ebola virus (West Africa, 2013) (21, 22) and the recent SARS-CoV2 (China, 2019) (23, 24). With shrinking habitats, caused by the Human population expansion, wild populations are co-existing in closer and closer proximity to humans leading to an increased threat of these events.

Studying the immune system of these species is key to assessing their resilience in a changing environment as well as what makes these unique mammals such good virus reservoirs. Still, the bats’ immune system is poorly understood. The innate immunity system (IIS) is the body’s first line of defence against pathogens. In this work, we analysed the *GBP* evolution in bats. We found a complex history of gene gain and loss with very different genetic repertoires between bat families.

## 2 Materials and methods

### 2.1 Phylogenetic analysis

Complete coding sequences of *GBP*s were obtained from publicly available databases. A total of 183 nucleotide sequences were collected from bats (129), primates (41), *Tupaia glis* (5) and *Loxodonta africana* (8). The accession numbers of these sequences can be found in **Supplementary Material: Table 1**. The *L. africana GBP* sequences were used as an outgroup. For bats, *GBP* sequences were collected only for species for which good-quality genomes are available at GenBank and Ensembl. Annotated GBP sequences were obtained through BLASTn searches using Human *GBP* sequences as queries. Searches were conducted in the NCBI’s GenBank (http://www.ncbi.nlm.nih.gov/genbank/) and Ensembl (https://www.ensembl.org/index.html) genome databases. Further BLASTn searches using Bat *GBP* sequences were conducted in both databases to ensure all bat *GBP* sequences were identified. In total, *GBP* sequences were obtained for 19 different species of bats belonging to eight families, Vespertilionidae, Miniopteridae, Phyllostomidae, Hipposideridae, Pteropodidae, Rhinolophidae, Mormoopidae and Molossidae.

Rodent GBP sequences, although available, were not used in this study. Muroid rodents GBP genes have a complex and seemingly specific pattern of evolution with four GBP genes that appear to be exclusive to Muroids (8). This diversity will add noise to our phylogenetic analysis causing a loss in resolution and correct identification of Bats GBPs.

Sequences were aligned with Clustal W (25) as implemented in BioEdit v7.2.5 (26), followed by visual inspection and necessary manual corrections. This dataset was screened for gene conversion using GARD (27); no recombination breakpoints were identified. The final nucleotide sequence alignment is given in **Supplementary Material: Data 1**.

The phylogenetic relationships between *GBP* nucleotide sequences were inferred using MEGA version 11 software (28) under a Maximum likelihood (ML) framework. The phylogenetic tree was constructed using the GTR+G+I model of nucleotide substitution, determined to be the best fitting model to our dataset by the Model Selection option in MEGA 11 (28). Node support was determined from 1000 bootstrap replicate trees.

### 2.2 Genomic synteny analysis

The relative syntenic positions and transcription orientation of bats *GBP*s were assessed using NCBI (https://www.ncbi.nlm.nih.gov/genome/gdv/).

### 2.3 Divergence analyses

**Figure 1:**
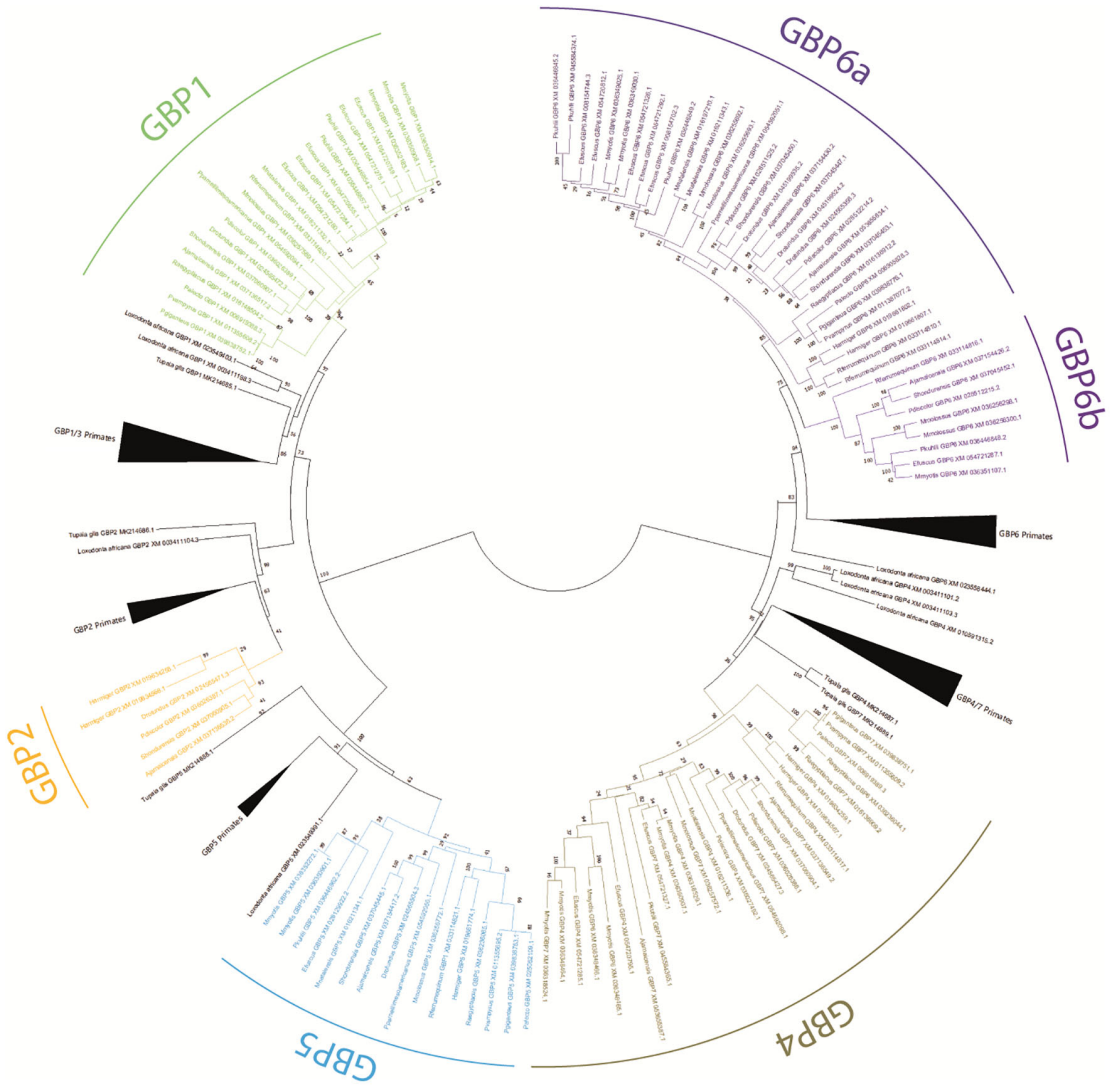
Phylogenetic tree of *GBP* genes in Chiroptera. A Maximum likelihood (ML) method and the GTR+G+I model of nucleotide substitution were used to obtain the *GBP* gene family phylogenetic tree. Bootstrap values are indicated near the most relevant branches.

Genetic distances between the groups established based on the ML tree (see **Figure 1**) were calculated using MEGA 11 (28). The net between group mean distances function was used to obtain the genetic distances between bat and primate *GBP* groups. This option accounts for variance due to differences within groups. These were calculated in MEGA 11 (28) software using the p-distance method, uniform rates among sites, homogeneous rates among lineages and pairwise deletion of gaps options.

The nucleotide substitution rate variation among the Chiroptera GBP6 genes was estimated in DnaSp version 6.12 (33). Sliding window analysis was performed with a window length of 250 nucleotides and a step size of 12 nucleotides along the nucleotide sequence alignment and plotting the differences as averages. Sites with alignment gaps were not counted.

## 3 Results

The obtained ML phylogenetic tree shows bats, primates, *Tupaia* and *L. africana GBP* sequences grouped according to gene with good bootstrap support (**Figure 1**). Sequences for bats *GBP1*, *GBP2*, *GBP4*, *GBP5*, and *GBP6* were identified. Within the *GBP4* cluster, several sequences annotated as *GBP6* and *GBP7* appear. These seem misannotated and need to be reclassified (see **Supplementary Material: Table 1**).

**Figure 2:**
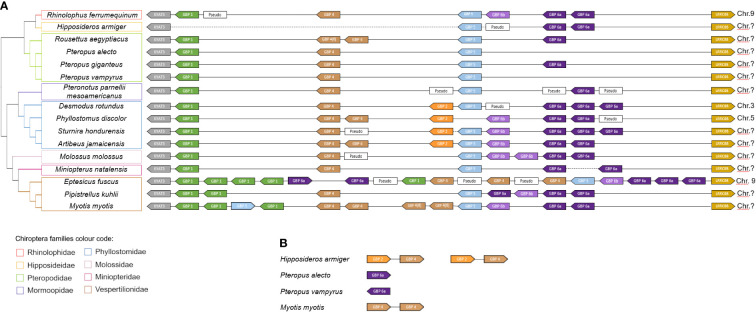
*GBP* gene family synteny in Chiroptera. **(A)** Organization of the *GBP* gene family in the studied species according to genomes available in NCBI (www.ncbi.nlm.nih.org). **(B)** Unplaced *GBP* in the bat genomes. The diagram is not drawn to scale. Arrows represent transcription orientation. Dashed lines represent gaps in the genome. Sequences excluded from the analysis for including stop codons or frameshifting indels are indicated as Pseudo. Chromosomes are indicated when information is available. Colour scheme: 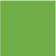 *GBP1*, 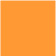 *GBP2*, 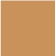 *GBP4*, 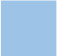 *GBP5*, 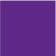 *GBP6a* and 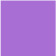 *GBP6b*.

Bat *GBP1* is present in all species except *Hipposideros armiger*. An incomplete sequence resembling *GBP*1 was found for this species, located where GBP1 would be expected, but it is not possible to confirm whether it is *GBP*1 or not. Most species have a single copy of *GBP1*. However, *Eptesicus fuscus* has five *GBP1* copies, *Myotis myotis* three *GBP1* genes and *Pippistrelus khuli* has two copies, showing that duplication events have occurred in Vespertilionidae bats (**Figures 1**, **2**).

Bat *GBP2* was identified only for species of *Phyllostomidae* and *Hipposideidae* bats. Considering that these sequences are clustering with the primate sequences and are present in both Yangochiroptera (*Phyllostomidae*) and Yinpterochiroptera (*Hipposideidae)* suborders (**Figures 1**, **2**), it suggests that *GBP2* was present in the Chiroptera ancestor and was lost independently in several lineages.

Bat *GBP4* and *GBP5* are present in all studied species, the exception being *Phyllostomus discolor* which has lost *GBP5*. For these two genes, *M. myotis* has suffered duplications, being the only studied bat species with more than one *GBP5* and having six *GBP4* genes, contrasting with most other species which have one or two *GBP4* copies (**Figure 1**, **Table 1**).

**Table 1:** Summary table showing the diversity of *GBP* genes found for each studied bat species.

Family	Species	Genes						Total
		*GBP1*	*GBP2*	*GBP4*	*GBP5*	*GBP6a*	*GBP6b*	
Rhinolophidae	*Rhinolophus ferrumequinum*	1	0	1	1	2	1	6
Hipposideidae	*Hipposideros armiger*	0	2	2	1	2	0	7
Pteropodidae	*Rousettus aegyptiacus*	1	0	2	1	1	0	5
	*Pteropus alecto*	1	0	1	1	1	0	4
	*Pteropus giganteus*	1	0	1	1	1	0	4
	*Pteropus vampyrus*	1	0	1	1	1	0	4
Mormoopidae	*Pteronotus parnellii mesoamericanus*	1	0	1	1	1	0	4
Phyllostomidae	*Desmodus rotundus*	1	1	1	1	3	0	7
	*Phyllostomus discolor*	1	1	2	0	2	1	7
	*Sturnira hondurensis*	1	1	1	1	3	1	8
	*Artibeus jamaicensis*	1	1	2	1	2	1	8
Molossidae	*Molossus molossus*	1	0	1	1	2	2	7
Miniopteridae	*Miniopterus natalensis*	1	0	1	1	2	0	5
Vespertilionidae	*Eptesicus fuscus*	5	0	3	1	5	1	15
	*Pipistrellus kuhlii*	2	0	1	1	3	1	8
	*Myotis myotis*	3	0	6	2	2	1	14
Total		23	6	26	17	33	9	114

Within the *GBP6* cluster, there are two well-supported subgroups (100 bootstrap for *GBP6b* and 85 bootstrap for *GBP6a*; **Figure 1**): a larger cluster containing sequences of the eight analysed bat families and a smaller cluster encompassing sequences for four of the analysed bat families. Within the larger cluster, most species have at least two copies of this *GBP6* gene, except for the species belonging to the Pteropodidae family which carry only one copy (**Figure 1**, **Table 1**). This is in contrast with the smaller cluster for which most species have only one copy of the gene, the exception being *Molossus molossus* with two copies (**Figure 1**, **Table 1**). This pattern suggests that the *GBP6* has duplicated in a Chiroptera ancestor originating two genes that have since followed distinct evolutionary patterns. Hereon, we shall designate the larger cluster as *GBP6a* and the smaller cluster as *GBP6b* (**Figure 1**).

It thus seems that the different bat *GBP*s have independently suffered deletions and/or duplications and are, thus, evolving under distinct evolutionary pressures. **Table 1** shows a summary of the number and repertoire found for each studied bat species. Pteropodidae species, having lost *GBP2* and *GBP6b*, have the least diversity of *GBP* genes and also the fewest copies since these have only one copy of each of the four other *GBP* genes present. The exception is *Rousettus aegyptiacus*, which has two copies of *GBP4*, resulting in five copies of *GBP* genes in *R. aegyptiacus* and four copies in the remaining Pteropodidae species (**Table 1**). In contrast, Vespertilionidae bats, in particular *M. Myotis* and *E. fuscus*, have an expansion of *GBP* genes, with a total of 14 and 15 *GBP* genes each, respectively (**Table 1**).

### 3.1 Synteny analysis

The bats’ *GBP* synteny is quite conserved (**Figure 2**). Despite the variability in the number of genes, bats’ *GBP*s are organized in tandem and flanked by *KYAT3* and *LRRC8B*. For *H. armiger*, *Pteropus alecto*, *Pteropus vampyrus* and *M. myotis*, unplaced *GBP* genes were found. For *H. armiger* a genome gap exists between KYAT3 and the GBP genes, not being possible to determine the full GBP locus organization for this species. The unplaced GBP2 and GBP4 genes may be located in the canonical GBP locus, between KYAT3 and GBP5, where these would be expected to be according to the other bats’ synteny. The *M. natalensis* and *S. hondurensis* assemblies also have one genome gap in each one. The *P. alecto*, *P. vampyrus* and *M. myotis* unplaced genes are most likely retrotransposons inserted in other genomic locations.

### 3.2 Divergence analysis

**Figure 3:**
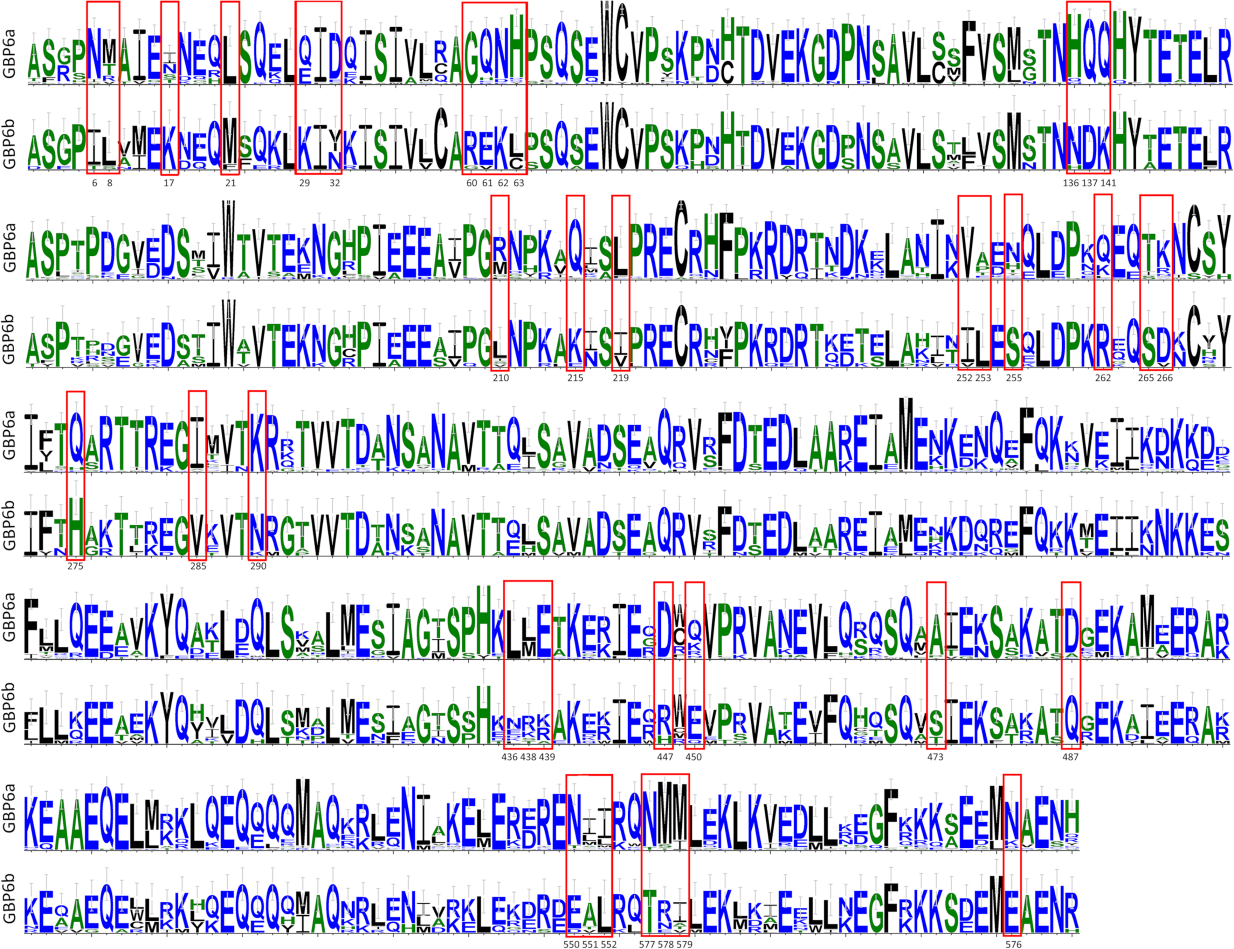
Bats Gbp6a and Gbp6b amino acid sequences diversity comparison. Alignments of the 33 Gbp6a and 9 Gbp6b sequences were used to create the sequence’s logo graphical representations, using the WebLogo program (29). Only amino acid variable positions are depicted (see **Supplementary Material: Data 3**). Amino acid residues that differentiate *GBP6a* from *GBP6b* are in red boxes; the position in the alignment for these residues is indicated below the boxes.

The genetic distances confirm that bat *GBP*s are orthologs to primate *GBP*s, with low divergence between bat *GBP*s and their primate counterpart (7%-10.2%; **Table 2**) and high divergence between bats’ *GBP*s (6.8-28.6%; **Table 2**). Considering that primates *GBP1* and *GBP3* are considered two genes with a divergence as low as 4%, the divergence of 6.8% between the two subgroups of bats’ *GBP6*, *GBP6a* and *GBP6b* (**Table 2**), supports their classification as different genes that arose from a duplication of *GBP6* in a bat ancestor. Furthermore, several amino acid residues that differentiate *GBP6a* from *GBP6b* are observable (**Figure 3**; see **Supplementary Material: Data 3**) and the amino acidic distance between these genes is high (11%). All the obtained results support the classification of *GBP6a* and *GBP6b* as different genes. This is considering 1) the good bootstrap support of the two bat *GBP6* groups in the ML phylogenetic tree, 2) their genetic distance being higher than that between p*GBP1* and p*GBP3* and 3) the existence of amino acid characteristic positions between *GBP6a* and *GBP6b*.

**Table 2:** Estimates of net evolutionary divergence between *GBP* groups of sequences. .

	Bats						Primates				
	*GBP1*	*GBP2*	*GBP4*	*GBP5*	*GBP6a*	*GBP6b*	p*GBP1*	p*GBP2*	p*GBP3*	p*GBP4*/*7*	p*GBP5*
*GBP2*	0.092										
*GBP4*	0.246	0.263									
*GBP5*	0.113	0.142	0.233								
*GBP6a*	0.251	0.265	0.092	0.247							
*GBP6b*	0.270	0.286	0.115	0.267	0.068						
p*GBP1*	0.083	0.119	0.288	0.149	0.288	0.308					
p*GBP2*	0.123	0.084	0.289	0.179	0.292	0.317	0.134				
p*GBP3*	0.080	0.118	0.285	0.150	0.291	0.309	0.043	0.127			
p*GBP4*/*7*	0.250	0.262	0.087	0.250	0.104	0.129	0.288	0.289	0.284		
p*GBP5*	0.174	0.204	0.300	0.102	0.309	0.332	0.202	0.223	0.197	0.313	
p*GBP6*	0.285	0.297	0.123	0.286	0.071	0.102	0.325	0.323	0.329	0.123	0.344

**Figure 4:**
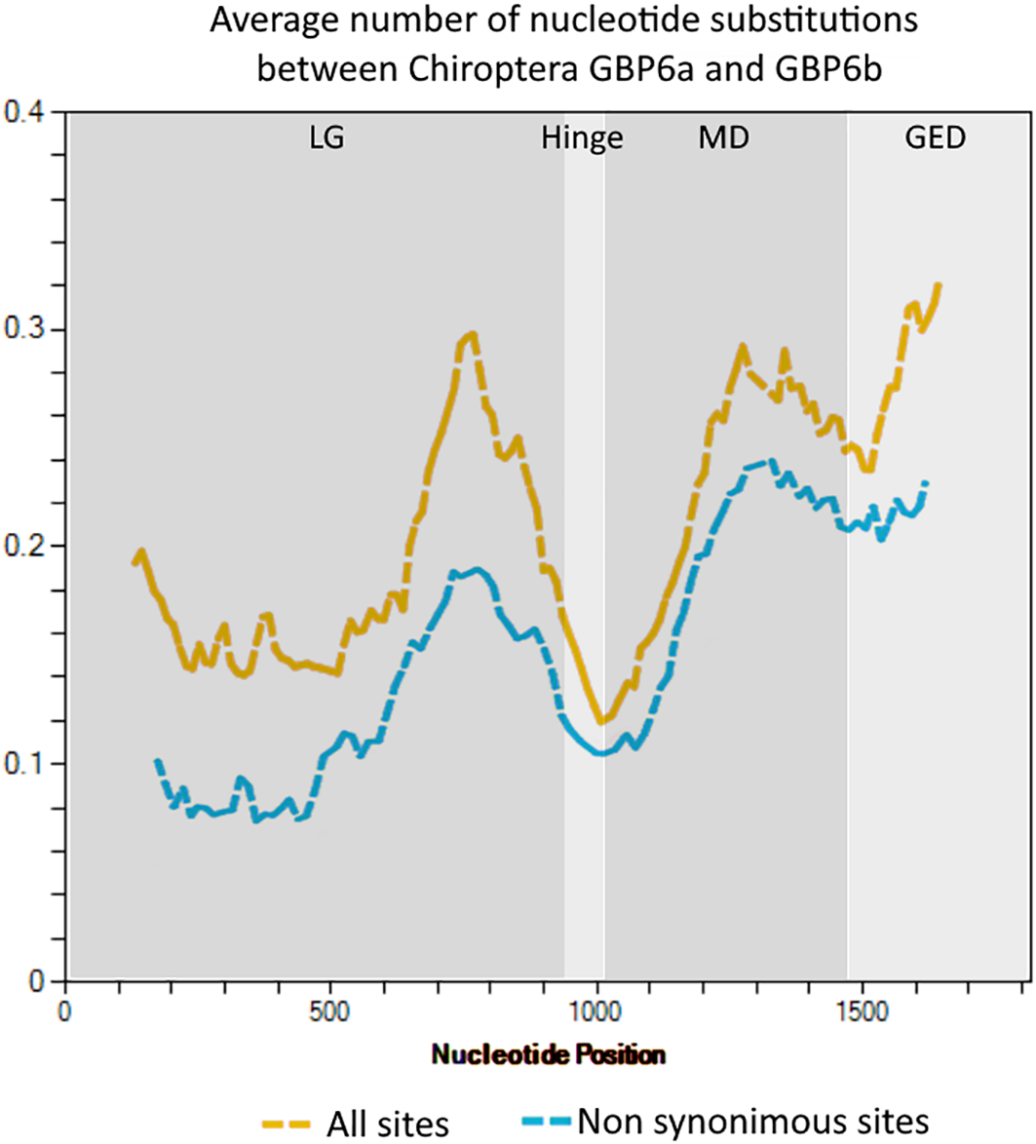
Sliding window analysis showing the nucleotide diversity along the *GBP6a* and *GBP6b* genes. The analysis was performed in DnaSP version 6.12 with a window length of 250 nucleotides and a step size of 12 nucleotides. The GBP domains are indicated.

The analysis of the nucleotide diversity along the two *GBP6* genes shows that, overall, this parameter is higher in the 3’ end of the LG, MD and GED domains. Focusing only on non-synonymous sites shows that a high proportion of the nucleotide substitutions between GBP6a and *GBP6b* are non-synonymous and confirms that the two genes’ amino acid sequence is very divergent, particularly in the effector regions of the genes (**Figure 4**; see **Supplementary Material: Data 2, 3**).

## 4 Discussion

Bats have been identified as a major reservoir for zoonotic viruses and have played roles in outbreaks of several emerging zoonotic viruses including SARS-CoV (China, 2002/2003) (17), Marburg virus (Africa, 2005) (18), MERS-CoV (Middle East, 2012) (19, 20), Ebola virus (West Africa, 2013) (21, 22) and the recent SARS-CoV2 (China, 2019) (23, 24). Viral reservoir species have an organized immunological response to the virus but show no overt clinical signs of disease. This means that the host usually carries a low viral load and is able to tolerate some viral replication. Studies have shown that bats have unique immunological approaches to enable coexistence with viral infections, causing no disease, while allowing enough viral replication for transmission (30–32). Screening the virome for over 4000 healthy bats from 40 different species of both Yangochiroptera and Yinpterochiroptera suborders, revealed an array of viruses belonging to diverse families, the most prevalent being Herpesviridae, Papillomaviridae, Retroviridae, Adenoviridae and Astroviridae, but also Coronaviridae, Caliciviridae, Polyomaviridae, Rhabdoviridae, among others (33), confirming bats as reservoirs of various virus. The unique ability of bats to act as reservoirs is thought to be mediated by a dampening of pro-inflammatory responses (30, 32, 34) as well as an increased resistance to infection mediated by special features of the antiviral type I interferon (IFN) system [reviewed in (35)]. *GBP*s are IFN-induced GTPases that have been shown to have vital roles in host immunity to infection and inflammation.

In this study, we screened available bat genomes for *GBP* genes. Sequences for bats *GBP1*, *GBP2*, *GBP4*, *GBP5*, and *GBP6* were identified, indicating that these could be orthologs to their human counterparts. It would be of interest to perform functional assays to confirm if functions remain conserved, for example, both human and mouse *GBP5* have been implicated in the NLRP3 activation upon bacterial infection (36). However, considering the obtained phylogenetic results, it is worth noting that some of the genes are poorly identified in the public databases. For example, some of the genes that are identified as *GBP4* or *GBP7* correspond, according to the presented phylogenetic tree, to *GBP6* genes. Furthermore, many genes in the *GBP4* group are annotated as *GBP7*. *GBP7*, however, has been shown to have emerged in primates as a duplication of p*GBP4* (9), our phylogenetic tree does not show a distinction between bats *GBP4* and *GBP7* and these are also phylogenetically close to *Tupaia glis* and *Loxodonta africana GBP4*. Accordingly, these genes should be classified as *GBP4*. For these reasons, we propose a review of the nomenclature of these genes in the aforementioned databases and greater attention in subsequent studies aiming to sequence genomes of bat species to avoid this type of error (see **Supplementary Material: Table 1**). Gene misannotation in public databases compromises not only evolutionary studies but also, and maybe more relevantly, the biological and functional understanding of the gene (37), leading to erroneous conclusions.

Interestingly, our analysis shows the existence of two *GBP6* genes in bats. The phylogenetic tree clustering (**Figure 1**), the calculated genetic distances (**Table 2**) and the existence of specific amino acids to both *GBP6a* and *GBP6b*, all support that these should be considered two independent genes. Furthermore, *GBP6a* and *GBP6b* seem to be evolving under different evolutionary pressures. The duplication of *GBP6* occurred in a Chiroptera ancestor, circa 62 million years ago (38) given that both *GBP6a* and *GBP6b* are present in Yinpterochiroptera and Yangochiroptera suborders. *GBP6a* has persisted in all studied species and suffered duplications in all but the Pteropodidae family species, *Pteropus* sp and *R. aegyptiacus*. *GBP6b*, on the contrary, has been lost independently in several lineages and is present mostly as a single-copy gene. These different evolutionary patterns and the existence of characteristic amino acids in each gene suggest that *GBP6a* and *GBP*6b have evolved or are evolving to perform different functions. All sequences present the four conserved elements for nucleotide binding and hydrolysis (see alignment in **Supplementary Material: Data 2**); G1-G4, where G1 (^45^GxxxxGKS/T^52^), G2 present a threonine (^75^T), G3 ^97^DxxG^100^ and G4 with ^179^T/AVRD^183^ (for *GBP4*, *6* and *7*) (39). Despite this, the amino acid differences between GBP6a and GBP6b are numerous. As such, functional studies should be performed to understand how these differences influence the biological roles of these two genes (expression, localization and role against pathogens).

Of worth noticing, is also the discrepancy in the number and diversity of *GBP* genes between bat families. Pteropodidae species, having lost *GBP2* and *GBP6b*, have the least diversity of *GBP* genes and also the fewest copies. On the opposite, Vespertilionidae bats, in particular *M. Myotis* and *E. fuscus*, have an expansion of *GBP* genes, each with a total of 15 *GBP* genes (**Table 1**). It is tempting to speculate that the loss of *GBP2* led to the expansion of *GBP4* in *M. Myotis* and *E. fuscus* since, in humans, upon *Salmonella* infection, *GBP1* requires *GBP2* and *GBP4* to recruit caspase-4 to the surface of the bacteria (2, 40). However, in *P. kuhlii* or Pteropodidae bats, this trend is not observed. The resulting patterns in the number of *GBPs* seem to be species-specific and could have been caused by host-pathogen co-evolution since bats can be reservoirs for several viruses. Gene expansions have been described for other immune system genes in Yangochiroptera bats, such as the *IFITM* locus (41), and more specifically in the genus M*yotis*, for which an unusual expansion of the *S100A7* genes occurred (42).

Although bat genomes are smaller than the genomes of other groups of mammals (13, 43), Pteropodidae (megabat) genomes tend to be smaller than the genomes of other bats (44). One of the reasons given is the fact that they have lost functions in an important line of long interspersed retrotransposable elements (LINES) known as LINE-1 (45). LINE-1 retrotransposons are the most abundant in mammals; in humans, for example, they account for around 15-20% of the genome (46). That said, it is possible that this and other similar events contributed to the notably reduced size of the megabat genome and more specific studies are needed to understand the reason for this reduction, as they are also the most distinct members of the chiropteran order, given their size, distribution, food, among others. The greater expansion of immune system genes in the genera *Myotis* and *Eptesicus* may be related to the success of these genera in colonizing new habitats (47).

This study further demonstrated that *GBP3* and *GBP7* are not present in bats, genes which are also absent in rodents (8). These results are congruent with the previous description of the emergence of these genes in primates, *GBP3* as a duplication of *GBP1* and *GBP7* as a duplication of *GBP4* (9). Several sequences of *GBP* pseudogenes were identified in the bat genomes (see synteny; **Figure 2A**), several genes were lost in different bat species and many *GBP* genes were duplicated. These data strongly support the birth-and-death model of evolution, which postulates that during evolution the genes from multigene families suffer duplications; some of these genes can be maintained in the genome, some can become pseudogenised and others can acquire a new function (3, 8, 9).

## 5 Conclusion

The results of this study show that several evolutionary processes occurred in the bats’ *GBP* gene family, such as gene deletions and duplications. These data are in accordance with the birth-and-death model of evolution, already attributed to members of this multigene family. An expansion of this gene family was also demonstrated in *M. myotis* and *E. fuscus*, and a reduction in it in members of the Pteropodidae family. A duplication of the *GBP6* gene was identified, which gave rise to two new genes, here named *GBP6a* and *GBP6b*. These genes present several different amino acids between the two genes, changes which may affect function; therefore, it is suggested that specific studies on the functions of these new genes should be carried out. Here, we also propose a review of the nomenclature of this gene family in order Chiroptera, since our results demonstrate that *GBP* genes in bats were poorly annotated. Additionally, each bat species presents a specific *GBP* evolution, possibly due to host-pathogen co-evolution. More evolutionary studies should be carried out to fully understand the complex evolution of *GBPs* and provide more insights into their function. Additionally, it will be important to sequence more bat species and improve the quality of some currently available genomes, so that more complete evolutionary studies can be carried out.

## Data availability statement

The datasets presented in this study can be found in online repositories. The names of the repository/repositories and accession number(s) can be found in the article/**Supplementary Material**.

## Author contributions

AP: Formal analysis, Methodology, Writing – original draft, Writing – review & editing. JB: Formal analysis, Writing – original draft, Writing – review & editing. JC-R: Formal analysis, Writing – review & editing. PE: Conceptualization, Funding acquisition, Supervision, Writing – review & editing.

## References

[1] Tretina K Park ES Maminska A MacMicking JD. Interferon-induced guanylate-binding proteins: Guardians of host defense in health and disease. J Exp Med (2019) 216(3):482–500. doi: 10.1084/jem.20182031 30755454 PMC6400534

[2] Kutsch M Coers J. Human guanylate binding proteins: nanomachines orchestrating host defence. FEBS J (2021) 288(20):5826–49. doi: 10.1111/febs.15662 PMC8196077 33314740

[3] Schelle L Corte-Real JV Esteves PJ Abrantes J Baldauf HM. Functional cross-species conservation of guanylate-binding proteins in innate immunity. Med Microbiol Immunol (2023) 212(2):141–52. doi: 10.1007/s00430-022-00736-7 PMC9005921 35416510

[4] Praefcke GJK. Regulation of innate immune functions by guanylate-binding proteins. Int J Med Microbiol (2018) 308(1):237–45. doi: 10.1016/j.ijmm.2017.10.013 29174633

[5] Li G Zhang J Sun Y Wang H Wang Y. The evolutionarily dynamic IFN-inducible GTPase proteins play conserved immune functions in vertebrates and cephalochordates. Mol Biol Evol (2009) 26(7):1619–30. doi: 10.1093/molbev/msp074 19369598

[6] Zhang R Li Z Tang YD Su C Zheng C. When human guanylate-binding proteins meet viral infections. J BioMed Sci (2021) 28(1):17. doi: 10.1186/s12929-021-00716-8 33673837 PMC7934404

[7] Olszewski MA Gray J Vestal DJ. In silico genomic analysis of the human and murine guanylate-binding protein (GBP) gene clusters. J Interferon Cytokine Res (2006) 26(5):328–52. doi: 10.1089/jir.2006.26.328 16689661

[8] Corte-Real JV Baldauf HM Melo-Ferreira J Abrantes J Esteves PJ. Evolution of guanylate binding protein (GBP) genes in muroid rodents (Muridae and cricetidae) reveals an outstanding pattern of gain and loss. Front Immunol (2022) 13:752186. doi: 10.3389/fimmu.2022.752186 35222365 PMC8863968

[9] Corte-Real JV Baldauf HM Abrantes J Esteves PJ. Evolution of the guanylate binding protein (GBP) genes: Emergence of GBP7 genes in primates and further acquisition of a unique GBP3 gene in simians. Mol Immunol (2021) 132:79–81. doi: 10.1016/j.molimm.2021.01.025 33550067

[10] Gu T Yu D Fan Y Wu Y Yao YL Xu L. Molecular identification and antiviral function of the guanylate-binding protein (GBP) genes in the chinese tree shrew (*Tupaia belangeri chinesis*). Dev Comp Immunol (2019) 96:27–36. doi: 10.1016/j.dci.2019.02.014 30817937

[11] Nei M Gu X Sitnikova T. Evolution by the birth-and-death process in multigene families of the vertebrate immune system. Proc Natl Acad Sci USA (1997) 94(15):7799–806. doi: 10.1073/pnas.94.15.7799 PMC33709 9223266

[12] Wandel MP Kim BH Park ES Boyle KB Nayak K Lagrange B. Guanylate-binding proteins convert cytosolic bacteria into caspase-4 signaling platforms. Nat Immunol (2020) 21(8):880–91. doi: 10.1038/s41590-020-0697-2 PMC7381384 32541830

[13] Teeling EC Vernes SC Davalos LM Ray DA Gilbert MTP Myers E. Bat biology, genomes, and the Bat1K project: To generate chromosome-level genomes for all living bat species. Annu Rev Anim Biosci (2018) 6:23–46. doi: 10.1146/annurev-animal-022516-022811 29166127

[14] Amador LI Moyers Arévalo RL Almeida FC Catalano SA Giannini NP. Bat systematics in the light of unconstrained analyses of a comprehensive molecular supermatrix. J Mamm Evolution. (2018) 25(1):37–70. doi: 10.1007/s10914-016-9363-8

[15] Shi JJ Rabosky DL. Speciation dynamics during the global radiation of extant bats. Evolution (2015) 69(6):1528–45. doi: 10.1111/evo.12681 25958922

[16] Agnarsson I Zambrana-Torrelio CM Flores-Saldana NP May-Collado LJ. A time-calibrated species-level phylogeny of bats (Chiroptera, mammalia). PloS Curr (2011) 3:RRN1212. doi: 10.1371/currents.RRN1212 21327164 PMC3038382

[17] Li W Shi Z Yu M Ren W Smith C Epstein JH. Bats are natural reservoirs of SARS-like coronaviruses. Science (2005) 310(5748):676–9. doi: 10.1126/science.1118391 16195424

[18] Amman BR Carroll SA Reed ZD Sealy TK Balinandi S Swanepoel R. Seasonal pulses of marburg virus circulation in juvenile rousettus aegyptiacus bats coincide with periods of increased risk of human infection. PloS Pathog (2012) 8(10):e1002877. doi: 10.1371/journal.ppat.1002877 23055920 PMC3464226

[19] Lau SKP Fan RYY Zhu L Li KSM Wong ACP Luk HKH. Isolation of MERS-related coronavirus from lesser bamboo bats that uses DPP4 and infects human-DPP4-transgenic mice. Nat Commun (2021) 12(1):216. doi: 10.1038/s41467-020-20458-9 33431849 PMC7801609

[20] Yang L Wu Z Ren X Yang F Zhang J He G. MERS-related betacoronavirus in vespertilio superans bats, china. Emerg Infect Dis (2014) 20(7):1260–2. doi: 10.3201/eid2007.140318 PMC4073873 24960574

[21] Mari Saez A Weiss S Nowak K Lapeyre V Zimmermann F Dux A. Investigating the zoonotic origin of the west african ebola epidemic. EMBO Mol Med (2015) 7(1):17–23. doi: 10.15252/emmm.201404792 25550396 PMC4309665

[22] Leroy EM Epelboin A Mondonge V Pourrut X Gonzalez JP Muyembe-Tamfum JJ. Human ebola outbreak resulting from direct exposure to fruit bats in luebo, democratic republic of congo, 2007. Vector Borne Zoonotic Dis (2009) 9(6):723–8. doi: 10.1089/vbz.2008.0167 19323614

[23] Wacharapluesadee S Tan CW Maneeorn P Duengkae P Zhu F Joyjinda Y. Evidence for SARS-CoV-2 related coronaviruses circulating in bats and pangolins in southeast asia. Nat Commun (2021) 12(1):972. doi: 10.1038/s41467-021-21240-1 33563978 PMC7873279

[24] Temmam S Vongphayloth K Baquero E Munier S Bonomi M Regnault B. Bat coronaviruses related to SARS-CoV-2 and infectious for human cells. Nature (2022) 604(7905):330–6. doi: 10.1038/s41586-022-04532-4 35172323

[25] Thompson JD Higgins DG Gibson TJ. CLUSTAL w: improving the sensitivity of progressive multiple sequence alignment through sequence weighting, position-specific gap penalties and weight matrix choice. Nucleic Acids Res (1994) 22(22):4673–80. doi: 10.1093/nar/22.22.4673 PMC308517 7984417

[26] Hall TA. BioEdit: a user-friendly biological sequence alignment editor and analysis program for windows 95/98/NT. Nucleic Acids Symposium Series. (1999) 41:3.

[27] Kosakovsky Pond SL Posada D Gravenor MB Woelk CH Frost SD. Automated phylogenetic detection of recombination using a genetic algorithm. Mol Biol Evol (2006) 23(10):1891–901. doi: 10.1093/molbev/msl051 16818476

[28] Tamura K Stecher G Kumar S. MEGA11: Molecular evolutionary genetics analysis version 11. Mol Biol Evolution. (2021) 38(7):3022–7. doi: 10.1093/molbev/msab120 PMC8233496 33892491

[29] Crooks GE Hon G Chandonia JM Brenner SE. WebLogo: A sequence logo generator. Genome Res (2004) 14(6):1188–90. doi: 10.1101/gr.849004 PMC419797 15173120

[30] Xie J Li Y Shen X Goh G Zhu Y Cui J. Dampened STING-dependent interferon activation in bats. Cell Host Microbe (2018) 23(3):297–301 e4. doi: 10.1016/j.chom.2018.01.006 29478775 PMC7104992

[31] Wiederkehr MA Qi W Schoenbaechler K Fraefel C Kubacki J. Virus diversity, abundance, and evolution in three different bat colonies in switzerland. Viruses (2022) 14(9):1911. doi: 10.3390/v14091911 36146717 PMC9505930

[32] Prescott J Guito JC Spengler JR Arnold CE Schuh AJ Amman BR. Rousette bat dendritic cells overcome marburg virus-mediated antiviral responses by upregulation of interferon-related genes while downregulating proinflammatory disease mediators. mSphere (2019) 4(6):e00728–19. doi: 10.1128/mSphere.00728-19 PMC6893212 31801842

[33] Wu Z Yang L Ren X He G Zhang J Yang J. Deciphering the bat virome catalog to better understand the ecological diversity of bat viruses and the bat origin of emerging infectious diseases. ISME J (2016) 10(3):609–20. doi: 10.1038/ismej.2015.138 PMC4817686 26262818

[34] Ahn M Anderson DE Zhang Q Tan CW Lim BL Luko K. Dampened NLRP3-mediated inflammation in bats and implications for a special viral reservoir host. Nat Microbiol (2019) 4(5):789–99. doi: 10.1038/s41564-019-0371-3 PMC7096966 30804542

[35] Zhang D Irving AT. Antiviral effects of interferon-stimulated genes in bats. Front Cell Infect Microbiol (2023) 13:1224532. doi: 10.3389/fcimb.2023.1224532 37661999 PMC10472940

[36] Shenoy AR Wellington DA Kumar P Kassa H Booth CJ Cresswell P. GBP5 promotes NLRP3 inflammasome assembly and immunity in mammals. Science (2012) 336(6080):481–5. doi: 10.1126/science.1217141 22461501

[37] Klimke W O'Donovan C White O Brister JR Clark K Fedorov B. Solving the problem: Genome annotation standards before the data deluge. Stand Genomic Sci (2011) 5(1):168–93. doi: 10.4056/sigs.2084864 PMC3236044 22180819

[38] Kumar S Stecher G Suleski M Hedges SB. TimeTree: A resource for timelines, timetrees, and divergence times. Mol Biol Evol (2017) 34(7):1812–9. doi: 10.1093/molbev/msx116 28387841

[39] Jimah JR Hinshaw JE. Structural insights into the mechanism of dynamin superfamily proteins. Trends Cell Biol (2019) 29(3):257–73. doi: 10.1016/j.tcb.2018.11.003 PMC9623552 30527453

[40] Santos JC Boucher D Schneider LK Demarco B Dilucca M Shkarina K. Human GBP1 binds LPS to initiate assembly of a caspase-4 activating platform on cytosolic bacteria. Nat Commun (2020) 11(1):3276. doi: 10.1038/s41467-020-16889-z 32581219 PMC7314798

[41] Scheben A Mendivil Ramos O Kramer M Goodwin S Oppenheim S Becker DJ. Long-read sequencing reveals rapid evolution of immunity- and cancer-related genes in bats. Genome Biol Evol (2023) 15(9):evad148. doi: 10.1093/gbe/evad148 37728212 PMC10510315

[42] Agueda-Pinto A Castro LFC Esteves PJ. The evolution of S100A7: an unusual gene expansion in myotis bats. BMC Evol Biol (2019) 19(1):102. doi: 10.1186/s12862-019-1433-0 31088346 PMC6518696

[43] Kapusta A Suh A Feschotte C. Dynamics of genome size evolution in birds and mammals. Proc Natl Acad Sci USA (2017) 114(8):E1460–E9. doi: 10.1073/pnas.1616702114 PMC5338432 28179571

[44] Smith JD Gregory TR. The genome sizes of megabats (Chiroptera: Pteropodidae) are remarkably constrained. Biol Lett (2009) 5(3):347–51. doi: 10.1098/rsbl.2009.0016 PMC2679926 19324635

[45] Cantrell MA Scott L Brown CJ Martinez AR Wichman HA. Loss of LINE-1 activity in the megabats. Genetics (2008) 178(1):393–404. doi: 10.1534/genetics.107.080275 18202382 PMC2206088

[46] Deininger PL Moran JV Batzer MA Kazazian HH Jr. Mobile elements and mammalian genome evolution. Curr Opin Genet Dev (2003) 13(6):651–8. doi: 10.1016/j.gde.2003.10.013 14638329

[47] Gunnell GF Smith R Smith T. 33 million year old myotis (Chiroptera, vespertilionidae) and the rapid global radiation of modern bats. PloS One (2017) 12(3):e0172621. doi: 10.1371/journal.pone.0172621 28273112 PMC5342209

