# Supplementary material for: Evolution of guanylate binding protein genes shows a remarkable variability within bats (Chiroptera)
Source: Front Immunol. 2024 Jan 31;15:1329098. doi: 10.3389/fimmu.2024.1329098 (PMC10864436; doi:10.3389/fimmu.2024.1329098)
Supplement: Supplementary file 1 [file DataSheet_1.zip › SuppMaterial/Supplementary Material_GBPs_revised.docx]

Supplementary Material

# Supplementary Data

**Supplementary Data 1.** Alignment of the Bats GBP nucleotide sequences used in this study.

**Supplementary Data 2.** Alignment of the Bats *GBP6a* and *GBP6b* nucleotide sequences.

**Supplementary Data 3.** Alignment of the Bats *GBP6a* and *GBP6b* amino acid sequences.

# Supplementary Tables

**Supplementary Table 1.** Genbank accession number of the sequences included and excluded in this study and proposed new nomenclature for Bats Gbp genes

**Included sequences**

| Accession number | *Species* | Gene name | Proposed classification |
| --- | --- | --- | --- |
| XM_033114820.1 | *Rhinolophus ferrumequinum* | *GBP 1* | *GBP 1* |
| XM_033114817.1 | *Rhinolophus ferrumequinum* | *GBP 4* | *GBP 4* |
| XM_033114821.1 | *Rhinolophus ferrumequinum* | *GBP 1* | *GBP5* |
| XM_033114810.1 | *Rhinolophus ferrumequinum* | *GBP 6* | *GBP 6a* |
| XM_033114814.1 | *Rhinolophus ferrumequinum* | *GBP 6* | *GBP 6a* |
| XM_033114816.1 | *Rhinolophus ferrumequinum* | *GBP 6* | *GBP 6b* |
| XM_019634258.1 | *Hipposideros armiger* | *GBP 2* | *GBP 2* |
| XM_019634566.1 | *Hipposideros armiger* | *GBP 2* | *GBP 2* |
| XM_019634259.1 | *Hipposideros armiger* | *GBP 4* | *GBP 4* |
| XM_019634567.1 | *Hipposideros armiger* | *GBP 4* | *GBP 4* |
| XM_019661774.1 | *Hipposideros armiger* | *GBP 5* | *GBP 5* |
| XM_019661802.1 | *Hipposideros armiger* | *GBP 6* | *GBP 6a* |
| XM_019661807.1 | *Hipposideros armiger* | *GBP 6* | *GBP 6a* |
| XM_016148504.2 | *Rousettus aegyptiacus* | *GBP 1* | *GBP 1* |
| XM_036236044.1 | *Rousettus aegyptiacus* | *GBP 6* | *GBP 4* |
| XM_016136909.2 | *Rousettus aegyptiacus* | *GBP 7* | *GBP 4* |
| XM_036236065.1 | *Rousettus aegyptiacus* | *GBP 5* | *GBP 5* |
| XM_016136912.2 | *Rousettus aegyptiacus* | *GBP 6* | *GBP 6a* |
| XM_006919388.3 | *Pteropus alecto* | *GBP 1* | *GBP 1* |
| XM_006919389.3 | *Pteropus alecto* | *GBP 7* | *GBP 4* |
| XM_025052109.1 | *Pteropus alecto* | *GBP 5* | *GBP 5* |
| XM_006905828.3 | *Pteropus alecto* | *GBP 6* | *GBP 6a* |
| XM_039838752.1 | *Pteropus giganteus* | *GBP 1* | *GBP 1* |
| XM_039838751.1 | *Pteropus giganteus* | *GBP 7* | *GBP 4* |
| XM_039838753.1 | *Pteropus giganteus* | *GBP 5* | *GBP 5* |
| XM_039838776.1 | *Pteropus giganteus* | *GBP 6* | *GBP 6a* |
| XM_011355608.2 | *Pteropus vampyrus* | *GBP 1* | *GBP 1* |
| XM_011355609.2 | *Pteropus vampyrus* | *GBP 7* | *GBP 4* |
| XM_011355695.2 | *Pteropus vampyrus* | *GBP 5* | *GBP 5* |
| XM_011387077.2 | *Pteropus vampyrus* | *GBP 6* | *GBP 6a* |
| XM_024565472.3 | *Desmodus rotundus* | *GBP 1* | *GBP 1* |
| XM_024565471.3 | *Desmodus rotundus* | *GBP 2* | *GBP 2* |
| XM_024565427.3 | *Desmodus rotundus* | *GBP 7* | *GBP 4* |
| XM_024565504.3 | *Desmodus rotundus* | *GBP 5* | *GBP 5* |
| XM_045199524.2 | *Desmodus rotundus* | *GBP 6* | *GBP 6a* |
| XM_045199535.2 | *Desmodus rotundus* | *GBP 6* | *GBP 6a* |
| XM_024565356.3 | *Desmodus rotundus* | *GBP 6* | *GBP 6a* |
| XM_036026389.1 | *Phyllostomus discolor* | *GBP 1* | *GBP 1* |
| XM_036026387.1 | *Phyllostomus discolor* | *GBP 2* | *GBP 2* |
| XM_036026386.1 | *Phyllostomus discolor* | *GBP 7* | *GBP 4* |
| XM_036027492.1 | *Phyllostomus discolor* | *GBP 4* | *GBP 4* |
| XM_028511525.2 | *Phyllostomus discolor* | *GBP 6* | *GBP 6a* |
| XM_028512214.2 | *Phyllostomus discolor* | *GBP 6* | *GBP 6a* |
| XM_028512215.2 | *Phyllostomus discolor* | *GBP 6* | *GBP 6b* |
| XM_037060907.1 | *Sturnira hondurensis* | *GBP 1* | *GBP 1* |
| XM_037060905.1 | *Sturnira hondurensis* | *GBP 2* | *GBP 2* |
| XM_037060904.1 | *Sturnira hondurensis* | *GBP 7* | *GBP 4* |
| XM_037045445.1 | *Sturnira hondurensis* | *GBP 5* | *GBP 5* |
| XM_037045447.1 | *Sturnira hondurensis* | *GBP 6* | *GBP 6a* |
| XM_037045450.1 | *Sturnira hondurensis* | *GBP 6* | *GBP 6a* |
| XM_037045453.1 | *Sturnira hondurensis* | *GBP 6* | *GBP 6a* |
| XM_037045452.1 | *Sturnira hondurensis* | *GBP 6* | *GBP 6b* |
| XM_037136517.2 | *Artibeus jamaicensis* | *GBP 1* | *GBP 1* |
| XM_037136536.2 | *Artibeus jamaicensis* | *GBP 2* | *GBP 2* |
| XM_037136549.2 | *Artibeus jamaicensis* | *GBP 7* | *GBP 4* |
| XM_037154417.2 | *Artibeus jamaicensis* | *GBP 5* | *GBP 5* |
| XM_037154430.2 | *Artibeus jamaicensis* | *GBP 6* | *GBP 6a* |
| XM_053655634.1 | *Artibeus jamaicensis* | *GBP 6* | *GBP 6a* |
| XM_037154426.2 | *Artibeus jamaicensis* | *GBP 6* | *GBP 6b* |
| XM_053655387.1 | *Artibeus jamaicensis* | *GBP 7 like* | *GBP 4* |
| XM_036257569.1 | *Molossus molossus* | *GBP 1* | *GBP 1* |
| XM_036257572.1 | *Molossus molossus* | *GBP 7* | *GBP 4* |
| XM_036256772.1 | *Molossus molossus* | *GBP 5* | *GBP 5* |
| XM_036255692.1 | *Molossus molossus* | *GBP 6* | *GBP 6a* |
| XM_036255693.1 | *Molossus molossus* | *GBP 6* | *GBP 6a* |
| XM_036256298.1 | *Molossus molossus* | *GBP 6* | *GBP 6b* |
| XM_036256300.1 | *Molossus molossus* | *GBP 6* | *GBP 6b* |
| XM_016211332.1 | *Miniopterus natalensis* | *GBP 1* | *GBP 1* |
| XM_016211336.1 | *Miniopterus natalensis* | *GBP 4* | *GBP 4* |
| XM_016211341.1 | *Miniopterus natalensis* | *GBP 5* | *GBP 5* |
| XM_016197210.1 | *Miniopterus natalensis* | *GBP 6* | *GBP 6a* |
| XM_016211343.1 | *Miniopterus natalensis* | *GBP 6* | *GBP 6a* |
| XM_054721280.1 | *Eptesicus fuscus* | *GBP 1* | *GBP 1* |
| *XM_054721275.1* | *Eptesicus fuscus* | *GBP 1 like* | *GBP 1* |
| *XM_054720759.1* | *Eptesicus fuscus* | *GBP 1* | *GBP 1* |
| *XM_054720655.1* | *Eptesicus fuscus* | *GBP 1 like* | *GBP 1* |
| *XM_054721284.1* | *Eptesicus fuscus* | *GBP 1 like* | *GBP 1* |
| *XM_054721326.1* | *Eptesicus fuscus* | *GBP 6 like* | *GBP 6a* |
| *XM_054721285.1* | *Eptesicus fuscus* | *GBP 4 like* | *GBP 4* |
| XM_054721327.1 | *Eptesicus fuscus* | *GBP 7 like* | *GBP 4* |
| XM_054720795.1 | *Eptesicus fuscus* | *GBP 4 like* | *GBP 4* |
| XM_028129922.2 | *Eptesicus fuscus* | *GBP 5* | *GBP 5* |
| XM_008154744.3 | *Eptesicus fuscus* | *GBP 6 like* | *GBP 6a* |
| XM_054720812.1 | *Eptesicus fuscus* | *GBP 6* | *GBP 6a* |
| XM_008154702.3 | *Eptesicus fuscus* | *GBP 6 like* | *GBP 6a* |
| XM_054721292.1 | *Eptesicus fuscus* | *GBP 6 like* | *GBP 6a* |
| XM_054721287.1 | *Eptesicus fuscus* | *GBP 6 like* | *GBP 6b* |
| XM_036446854.2 | *Pipistrellus kuhlii* | *GBP 1* | *GBP 1* |
| XM_036446857.2 | *Pipistrellus kuhlii* | *GBP 1* | *GBP 1* |
| XM_045584365.1 | *Pipistrellus kuhlii* | *GBP 7* | *GBP 4* |
| XM_036446862.2 | *Pipistrellus kuhlii* | *GBP 5* | *GBP 5* |
| XM_036446845.2 | *Pipistrellus kuhlii* | *GBP 6* | *GBP 6a* |
| XM_045584374.1 | *Pipistrellus kuhlii* | *GBP 6* | *GBP 6a* |
| XM_036446849.2 | *Pipistrellus kuhlii* | *GBP 6* | *GBP 6a* |
| XM_036446848.2 | *Pipistrellus kuhlii* | *GBP 6* | *GBP 6b* |
| XM_036352183.1 | *Myotis myotis* | *GBP 1* | *GBP 1* |
| XM_036350908.1 | *Myotis myotis* | *GBP 1* | *GBP 1* |
| XM_036350914.1 | *Myotis myotis* | *GBP 1* | *GBP 1* |
| XM_036318529.1 | *Myotis myotis* | *GBP 4* | *GBP 4* |
| XM_036350907.1 | *Myotis myotis* | *GBP 4* | *GBP 4* |
| XM_036348465.1 | *Myotis myotis* | *GBP 6* | *GBP 4* |
| XM_036348466.1 | *Myotis myotis* | *GBP 6* | *GBP 4* |
| XM_036348464.1 | *Myotis myotis* | *GBP 4* | *GBP 4* |
| XM_036318524.1 | *Myotis myotis* | *GBP 7* | *GBP 4* |
| XM_036350901.1 | *Myotis myotis* | *GBP 5* | *GBP 5* |
| XM_036352272.1 | *Myotis myotis* | *GBP 5* | *GBP 5* |
| XM_036349025.1 | *Myotis myotis* | *GBP 6* | *GBP 6a* |
| XM_036349030.1 | *Myotis myotis* | *GBP 6* | *GBP 6a* |
| XM_036351107.1 | *Myotis myotis* | *GBP 6* | *GBP 6b* |
| XM_054592094.1 | *Pteronotus parnellii mesoamericanus* | *GBP 1 like* | *GBP 1* |
| XM_054592098.1 | *Pteronotus parnellii mesoamericanus* | *GBP 7 like* | *GBP 4* |
| XM_054592051.1 | *Pteronotus parnellii mesoamericanus* | *GBP 6 like* | *GBP 6a* |
| XM_054592050.1 | *Pteronotus parnellii mesoamericanus* | *GBP 5 like* | *GBP 5* |
| XM_012464810.1 | *Aotus nancymaae* | *GBP 1* | n.a. |
| XM_012464814.1 | *Aotus nancymaae* | *GBP 2* | n.a. |
| XM_012464809.2 | *Aotus nancymaae* | *GBP 3* | n.a. |
| XM_012464811.1 | *Aotus nancymaae* | *GBP 4* | n.a. |
| XM_012464815.2 | *Aotus nancymaae* | *GBP 5* | n.a. |
| XM_012464820.2 | *Aotus nancymaae* | *GBP 6* | n.a. |
| XM_012464776.1 | *Aotus nancymaae* | *GBP 6* | n.a. |
| XM_012464812.1 | *Aotus nancymaae* | *GBP 7* | n.a. |
| XM_011956051.1 | *Colobus angolensis palliatus* | *GBP 1* | n.a. |
| XM_011956052.1 | *Colobus angolensis palliatus* | *GBP 2* | n.a. |
| XM_011956056.1 | *Colobus angolensis palliatus* | *GBP 3* | n.a. |
| XM_011956049.1 | *Colobus angolensis palliatus* | *GBP 6* | n.a. |
| XM_011956192.1 | *Colobus angolensis palliatus* | *GBP 6* | n.a. |
| XM_011956054.1 | *Colobus angolensis palliatus* | *GBP 7* | n.a. |
| XM_019026669.3 | *Gorilla gorilla gorilla* | *GBP 1* | n.a. |
| XM_019026761.3 | *Gorilla gorilla gorilla* | *GBP 2* | n.a. |
| XM_004026097.4 | *Gorilla gorilla gorilla* | *GBP 3* | n.a. |
| XM_019027006.3 | *Gorilla gorilla gorilla* | *GBP 4* | n.a. |
| XM_004026101.3 | *Gorilla gorilla gorilla* | *GBP 5* | n.a. |
| XM_004026102.3 | *Gorilla gorilla gorilla* | *GBP 6* | n.a. |
| XM_019027091.3 | *Gorilla gorilla gorilla* | *GBP 7* | n.a. |
| NM_002053.3 | *Homo sapiens* | *GBP 1* | n.a. |
| NM_004120.5 | *Homo sapiens* | *GBP 2* | n.a. |
| BC140837.1 | *Homo sapiens* | *GBP 3* | n.a. |
| NM_052941.5 | *Homo sapiens* | *GBP 4* | n.a. |
| NM_052942.5 | *Homo sapiens* | *GBP 5* | n.a. |
| BC131713.1 | *Homo sapiens* | *GBP 6* | n.a. |
| NM_207398.3 | *Homo sapiens* | *GBP 7* | n.a. |
| XM_001085311.4 | *Macaca mulatta* | *GBP 1* | n.a. |
| XM_001085895.4 | *Macaca mulatta* | *GBP 2* | n.a. |
| XM_001083783.4 | *Macaca mulatta* | *GBP 3* | n.a. |
| XM_015144556.2 | *Macaca mulatta* | *GBP 6* | n.a. |
| XM_015144533.2 | *Macaca mulatta* | *GBP 6* | n.a. |
| XM_015144520.2 | *Macaca mulatta* | *GBP 7* | n.a. |
| XM_001147994.6 | *Pan troglodytes* | *GBP 1* | n.a. |
| XM_024346929.2 | *Pan troglodytes* | *GBP 2* | n.a. |
| XM_001146987.5 | *Pan troglodytes* | *GBP 3* | n.a. |
| XM_009424854.4 | *Pan troglodytes* | *GBP 4* | n.a. |
| XM_016922023.3 | *Pan troglodytes* | *GBP 5* | n.a. |
| XM_016922096.3 | *Pan troglodytes* | *GBP 6* | n.a. |
| XM_009424758.4 | *Pan troglodytes* | *GBP 7* | n.a. |
| MK214685.1 | *Tupaia glis* | *GBP 1* | n.a. |
| MK214686.1 | *Tupaia glis* | *GBP 2* | n.a. |
| MK214687.1 | *Tupaia glis* | *GBP 4* | n.a. |
| MK214688.1 | *Tupaia glis* | *GBP 5* | n.a. |
| MK214689.1 | *Tupaia glis* | *GBP 7* | n.a. |
| XM_023549396.1 | *Loxodonta africana* | *GBP 1* | n.a. |
| XM_003411198.3 | *Loxodonta africana* | *GBP 1* | n.a. |
| XM_023549403.1 | *Loxodonta africana* | *GBP 1* | n.a. |
| XM_003411104.3 | *Loxodonta africana* | *GBP 2* | n.a. |
| XM_003411101.2 | *Loxodonta africana* | *GBP 4* | n.a. |
| XM_003411103.3 | *Loxodonta africana* | *GBP 4* | n.a. |
| XM_010591315.2 | *Loxodonta africana* | *GBP 4* | n.a. |
| XM_023549391.1 | *Loxodonta africana* | *GBP 5* | n.a. |

**Excluded sequences**

| **Accession number** | **Species** | **Gene name** | **Reason for exclusion** |
| --- | --- | --- | --- |
| XM_019661775.1 | *Hipposideros armiger* | *GBP 6* | partial mRNA |
| XM_019661773.1 | *Hipposideros armiger* | *GBP 6 like* | Premature stop codons |
| LOC109376723 | *Hipposideros armiger* | *uncharacterized* | Partial mRNA |
| XM_036027493.1 | *Phyllostomus discolor* | *GBP 6* | Short sequence |
| XM_036256773.1 | *Molossus molossus* | *GBP 7* | Frameshifting indels |
| XM_016197215.1 | *Miniopterus natalensis* | *GBP 6* | Short sequence |
| XM_054721325.1 | *Eptesicus fuscus* | *GBP6 like* | Short sequence |
| XM_054720653.1 | *Eptesicus fuscus* | *GBP 7 like* | Premature stop codons |
| XM_054721286.1 | *Eptesicus fuscus* | *GBP4 like* | Premature stop codons |
| XM_036349031.1 | *Myotis myotis* | *GBP 6* | Short sequence |
| XM_036318523.1 | *Myotis myotis* | *GBP 1* | partial mRNA |
| XM_036350913.1 | *Myotis myotis* | *GBP 2* | Short sequence |
| XM_036348463.1 | *Myotis myotis* | *GBP1 like* | Short sequence |
| XM_054592049.1 | *Pteronotus parnellii mesoamericanus* | *GBP 2 like* | Premature stop codons |
| XM_054592178.1 | *Pteronotus parnellii mesoamericanus* | *GBP 6 like* | Premature stop codons |
| LOC129085630 | *Pteronotus parnellii mesoamericanus* | *GBP Pseudogene* | *Pseudogene* |
| XM_037061010.1 | *Sturnira hondurensis* | *GBP 7 like* | Frameshifting indels |
| LOC112309271 | *Desmodus rotundus* | *GBP Pseudogene* | *Pseudogene* |
| LOC129150319 | *Eptesicus fuscus* | *GBP1 Pseudogene* | *Pseudogene* |
